# A Case of Gouty Peripheral Neuritis

**Published:** 1888-03

**Authors:** F. W. Jollye

**Affiliations:** Warminster, Wilts


### Clinical Records.

### A CASE OF GOUTY PERIPHERAL NEURITIS.

By F. W. Jollye, M.R.C.S., L.R.C.P. Lond., Warminster, Wilts.

This case occurred in the practice of Mr. R. L. Willcox, to whose kindness I am indebted for permission to publish these notes.

G. W., æt. 61, a publican, but never a hard drinker, and with no history of syphilis, had been since the middle of 1885 liable to frequent attacks of renal colic. For several years previously he had had occasional attacks of gout in his feet; during these attacks of colic he would pass quantities of small uric acid calculi.

During the early part of the year 1886, he had several attacks of severe pleurodynia—at first limited to the right side, but afterwards being confined entirely to the left. His urine was of low sp. gr. and contained traces of albumen, and on one occasion granular casts were discovered. His pulse was of high tension, the aortic sound being accentuated and his heart slightly hypertrophied.

In the autumn of 1886 he began to complain of sharp shooting neuralgic pains down the course of the right sciatic and anterior crural nerves, and of "pins and needles" in the right foot; these pains were worse at night, and would wake him up, and there was distinct tenderness on pressure over the course of these nerves. After two months the left leg became similarly affected; and sudden spasms of the hamstrings would occur, drawing his knees up in bed and causing him great pain: these spasms were increased in frequency and severity after walking. He was also tender on pressure over the posterior branches of the lumbar and sacral plexuses on each side of the spinal column, but there was no pain on percussion over the spinous processes. The muscles of the legs were all more or less tender on manipulation. He could stand with his heels together and his eyes closed, and could feel the ground perfectly with his feet. The left knee-jerk was quite gone, while the right one was still fairly marked; the left plantar reflex was less marked than the right. His legs below the knee were œdematous, especially the left one. His bowels and bladder acted normally, but he suffered a good deal from tympanites. He had no sickness.

In January, 1887, there was noticeable wasting of the extensors of both legs, and also of the vasti, but more apparent on the left side; the flexors and hamstrings were also wasted and very flaccid. The fulgurating pains were also marked, but he would at times have almost complete freedom from them for one or two days. When sitting he could not lift the toes of the left foot off the ground, and when lying down he could not lift his left heel off the bed; but these movements were nearly perfect on the right side. The transmission of sensory stimuli was delayed on the left side, but the appreciation of cold and warmth was normal. The skin of the lower two-thirds of the legs became destitute of hairs, and shiny.

In March he complained of "pins and needles" in the fingers, first of his right hand and soon in his left: these pains were more acute in the ring and little fingers than in the remainder; there was marked tenderness of the ulnar nerves above the elbow, where they could be distinctly felt as if enlarged, and the least pressure caused shooting pains down his forearm and fingers. There was a varying tenderness in the interscapular region and over several of the intercostal spaces. The muscles of the forearms soon began to atrophy, and the hand-grasps—especially of the right one—were very feeble; the mucles of the hands being slightly wasted.

In June he suddenly had an attack of gout in the great toe of the right foot, which lasted about seven days, during which time he was almost free from any pain; but it returned as soon as the attack was over.

In July he complained of a throbbing headache, limited to the right side. This side of his head was distinctly tender; and the right pupil was smaller than the left, but both reacted to light and accommodation. In a few days there was a distinct thickening just in front of the right parietal eminence, which was tender and elastic. He gradually became feebler, the muscular wasting was more marked, the biceps and triceps of the arms likewise atrophied, and in October he was completely confined to his bed. At this time the electrical reaction of his muscles was examined, and all the muscles of the four extremities gave the reaction of degeneration, but this was more decided in the extensors and in the muscles nearest the periphery than in the flexors and muscles nearer the trunk.

In November the left knee-jerk returned slightly; the muscles of the leg seemed to be increasing in size, and the pain was decidedly less; but now the right side of the tongue became swollen and painful, the food began to collect between the right cheek and lower jaw, and his speech was muffled. In about three weeks, the right side of his tongue became smaller than the left, and protruded towards the right, and he could not touch the left corner of his mouth with the tip of his tongue, but the muscles of the left half of the tongue seemed to act normally. About the same time, symptoms of right facial paralysis began to develop around the mouth, but the right orbicularis palpebrarum acted normally.

When seen on December 6th, he said he had not passed water for nearly forty-eight hours, but was in no pain; but on examination the bladder was very distended, and over a quart of urine was drawn off by the catheter. After this he could never tell when his bladder was distended. In a few days cystitis set in, and until his death, on December 25th, his urine dribbled from him and became horribly offensive, being mixed with pus, mucus, and blood; and although he was given large doses of boric acid, it had no effect on the putrefaction of the urine. His tongue became dry and brown, his breath offensive, and he gradually died from septicæmia caused by the cystitis. No post-mortem was allowed.

From the beginning to the end of his illness, his mental faculties were normal, and he had no bedsores, nor any trophic lesions of the skin except the loss of hair before mentioned.

About a week before his death his pulse became intermittent for one day, but soon after the commencement of his illness he lost all signs of high arterial tension.

His temperature was taken during a few of his attacks of pleurodynia, and was found to be raised about 1°; but during the last few months of his illness the temperature was always below normal, once sinking as low as 96.8° in the axilla.

*Treatment.*—He was treated at first with the ordinary gouty remedies; viz., alkalies, saline aperients, colchicum and iodide of potassium.

His diet consisted chiefly of milk and farinaceous food, with a little beef-tea and mutton, and no stimulants at first; but as no improvement took place after several months of this treatment, and he was gradually getting weaker, he was ordered iron and quinine, with cod-liver oil, and allowed a little whisky.

For the relief of pain he was given chloral hydrate and bromide of potassium, and afterwards tinct. of cannabis indica; but these did not secure freedom from pain, and so, notwithstanding the state of his kidneys, he was given opium in sufficient doses to give him ease and sleep during the last nine months of his illness. On several occasions, when the lightning pains were very severe, he had hypodermic injections of morphia (gr. $\frac{1}{2}$).

To relieve the throbbing headache, an ointment of belladonna and lanoline was smeared on that side of the head, and a mixture of ergot and digitalis given. The digitalis lowered his pulse-rate to 60 per minute, and, while it kept down, his head was relieved but it never seemed to cause contraction of the peripheral arterioles and so to raise the blood-pressure; nor was the quantity of urine excreted increased: perhaps this was due to a more or less vaso-motor paralysis.

The prognosis in these cases of advanced gouty peripheral neuritis seems to be less hopeful than those of alcoholic, lead, or diphtheritic origin; and the ordinary gouty remedies in this case, as in the two recorded by Graves in his *Clinical Medicine*, seemed to have no effect on the course of the disease.

*Remarks.*—This appears to be a case of gouty, and not alcoholic paralysis; for, although formerly he used to drink rather more beer than was good for him, after his first attack of renal colic he became almost a total abstainer.

This case resembles one described by Dr. Graves* in which the onset manifested itself by attacks of pleurodynia, and in the fact that his patient was free from pain during an attack of acute gout.† In Dr. Graves's case there was also retention of urine, as there was also in a case recorded by Dr. Grainger Stewart,‡ and in another one related by Dr. Buzzard; § but in none of these cases is it stated that it was "painless retention." Mr. J. Hutchinson has called attention to a state of retention, which he called "painless retention of urine," || and which he has found chiefly in connection with two conditions—senility and locomotor ataxy; and he goes on to say—"It is in connection with ataxy that the best marked and most absolutely painless cases are met. It must be borne in mind, in this connection, that old age and ataxy have many symptoms in common; that an obtunding of sensibility, and especially of certain forms of it, is a very frequent condition in the aged. In the painless retention which we encounter in connection with ataxy, however, the symptom is a most definite and extraordinary one. The bladder, not being the seat of any previous hypertrophy, is capable of great dilatation, and it may fill until it reaches the umbilicus without any overflow occurring, and without the slightest pain; sometimes a sense of mechanical fulness is present, but there

* Graves's *Clinical Medicine*, vol i., p. 551. † *Ibid.*, p. 552.

‡ *Edin. Med. Jour.*, April, 1881, referred to by Dr. Buzzard in *Paralysis from Peripheral Neuritis*, p. 51.

§ *Paralysis from Peripheral Neuritis*, by Dr. Buzzard, p. 74.

|| *British Medical Journal*, March 12th, 1887.

is nothing approaching pain. I have several times known ataxic patients retain their urine for more than forty-eight hours without any suffering. Whenever the bladder shows unusual tolerance of distension, the symptoms of ataxy should be searched for."

In this case the patient only complained that he had not passed water for some time, and was quite surprised when he was told that his bladder was distended. This painless retention was due no doubt to anæsthesia of the walls of the bladder, due to a peripheral neuritis of the fibres of the posterior branches of the 3rd, 4th, and 5th sacral nerves which supply sensation to the walls of the bladder. According to Landois,* if the arc of the bladder-reflex is destroyed, voluntary micturition is impossible; for he says the smooth musculature of the bladder cannot be excited directly by a voluntary impulse, but is always caused to contract reflexly.

Dr. Graves considered that retention of urine shows that the mischief has spread to the spinal cord. In his case above referred to, the spinal cord, from the 3rd dorsal vertebra downwards, was of a yellowish hue; and he states that retention of urine in this disease differs from that which is due to primary disease of the spinal cord in being one of the late symptoms, coming on after the paralysis has crept up from the extremities along the nerves towards the spinal marrow; whereas in primary disease of the cord, retention of urine or irritability of the bladder often announces the approach of the disease long before the loss of power in the limbs becomes evident.†

As is usual in these cases, there were marked vaso-motor disturbances, as shown by the œdema of the legs and the primary swelling of the right side of the tongue so

* Landois and Stirling's *Physiology*, p. 653. † Graves's *Clinical Medicine*, p. 555.

that it felt "too big for his mouth." The very distressing throbbing headache was also alone felt on this side, showing that the vaso-motor nerves of the head were affected unilaterally. Dr. Buzzard has suggested that peripheral neuritis is due to a toxic influence of some kind exerted upon vaso-motor centres in the bulb and cord, leading to spasm of the arterioles, and thus diminishing the supply of blood to the peripheral nerves.* The same author mentions a case of diphtheritic paralysis in which one side of the tongue was atrophied and the muscles of the lips were more or less paralysed, and points out that the limitation of the facial paresis to the muscles about the mouth points rather to lesion of a nerve than of its centre.†

A neuritis of the pneumogastric was not the cause of death in this case, although it might have been affected; for he once had an intermittent pulse, and occasionally had attacks of dyspnœa, but the mode of death resembled a toxic one more than any other.

The symptoms in favour of the diagnosis of peripheral neuritis, summed up, are the following:

1. The pain remained limited to one side for some time before suddenly appearing on the other.
2. The lightning-like pains in the extremities, and the "pins and needles" in the fingers and toes.
3. The marked tenderness of several nerve-trunks.
4. The hyperæsthesia and wasting of the muscles of the extremities, accompanied by the R.D.
5. The vaso-motor disturbances.
6. The paralysis beginning in extensors and spreading towards the trunk, and afterwards affecting to a slighter extent the hands and arms.

* *Paralysis from Peripheral Neuritis*, p. 56. † *Ibid.*, pp. 119, 120.

7. The decided intermissions of pain which he frequently enjoyed during the ealier stages of the disease.
8. The relief from pain during the attack of gout.
9. The retention of urine coming on as a very late symptom.
10. No affection of the mental faculties.
11. Absence of bedsores.

---

